# Analysis of Stress-Responsive Transcriptome in the Intestine of Asian Seabass (*Lates calcarifer*) using RNA-Seq

**DOI:** 10.1093/dnares/dst022

**Published:** 2013-06-10

**Authors:** Jun Hong Xia, Peng Liu, Feng Liu, Grace Lin, Fei Sun, Rongjian Tu, Gen Hua Yue

**Affiliations:** 1 Molecular Population Genetics Group, Temasek Life Sciences Laboratory, 1 Research Link, National University of Singapore, Singapore117604, Singapore; 2 Department of Biological Sciences, National University of Singapore, 14 Science Drive 4, Singapore 117543, Singapore

**Keywords:** RNA-seq, intestine, stress, disease, nutrition

## Abstract

Identification of differentially expressed genes (DEGs) and regulated pathways in response to stressors using a whole-genome approach is critical to understanding the mechanisms underlying stress responses. We challenged Asian seabass with lipopolysaccharide (LPS), *Vibrio harveyi*, high salinity and fasting, and sequenced six cDNA libraries of intestine samples using Roche 454 RNA-seq. Over 1 million reads (average size: 516 bp) were obtained. The *de novo* assembly obtained 83 911 unisequences with an average length of 747 bp. In total, 62.3% of the unisequences were annotated. We observed overall similar expression profiles among different challenges, while a number of DEGs and regulated pathways were identified under specific challenges. More than 1000 DEGs and over 200 regulated pathways for each stressor were identified. Thirty-seven genes were differentially expressed in response to all challenges. Our data suggest that there is a global coordination and fine-tuning of gene regulation during different challenges. In addition, we detected dramatic immune responses in intestines under different stressors. This study is the first step towards the comprehensive understanding of the mechanisms underlying stress responses and supplies significant transcriptome resources for studying biological questions in non-model fish species.

## 1. Introduction

Stress response is important to living organisms and a major aspect of natural selection in the wild.^1^ Fishes are exposed to a variety of stressors because their homeostatic mechanisms are highly dependent on prevailing conditions primarily related to the aquatic environment.^2^ Previous studies showed that the interactions between stressors and stress responses are complex.^2^ Genetic, developmental and environmental factors, and early experiences have influences on the stress responses of fish.^3,4^ Identification of differentially expressed genes (DEGs) and pathways under stress conditions is important for the understanding of fish stress response. A lot of platforms to reveal DEGs are available, such as real-time (RT)-PCR and microarray. Recently, RNA-Seq^5^ is considered to be a revolutionary tool for transcriptomics, as it can absolutely quantify over millions of unknown transcripts. It has also shown a great analytical power in the identification of differentially expressed transcripts in response to different conditions.^6–9^ For species with poor genome annotation, the Roche/454-based RNA-seq technique with much longer read lengths provides an excellent tool for expression profiling studies.^10,11^

The intestine is one of the major organs in fish that interact with the environment and is involved in many biological processes. A few studies on the responses of the intestine to various stressors have been performed in fishes, such as medaka^12^ and channel catfish.^13^ However, data on responses of the fish intestine to different stresses are still limited. Studies of the genome-wide transcriptome after challenging with different stressors in fish will enhance our understanding of the molecular mechanisms underlying stresses.

The Asian seabass (*Lates calcarifer*) is an important food fish species in Southeast Asia and Australia.^14^ Although some expressed sequence tags (ESTs) of the Asian seabass are available in public domains,^15–17^ the transcriptome for the Asian seabass is still poorly characterized. To date, an intestine transcriptome data set for the Asian seabass and most food fish species is lacking. To gain more understanding of the mechanisms underlying stress responses and to supply significant transcriptome resources for studying biological questions in non-model fish species, we conducted RNA-seq of intestine samples from fishes challenged with lipopolysaccharide (LPS), *Vibrio harveyi* infection, fasting and high salinity. We also carried out bioinformatic analyses of the transcriptome to identify DEGs and pathways in response to these different stressors.

## 2. Materials and methods

### 2.1. Fish management, challenges and sampling for RNA-seq analysis

Thirty-six Asian seabass at the age of 11 months (body weight ∼330 g) were originally maintained in a large tank containing 2000 l of freshwater in the animal outhouse of our institute. For challenge experiments, 12 fishes were transferred to a 1000-l tank, and the salinity concentration was gradually increased to full-seawater (33 PPT salinity) within 3 days. Fishes were fed twice daily with pelleted feed (Biomar, Nersac, France). One day prior to challenges, nine seabass from the seawater tank, after acclimatization for 2 weeks, were evenly divided into three tanks containing 300 l of seawater (i.e. 3 fishes per tank). For the three fishes in tank 1, named as Int1 (LPS), each fish was injected intra-peritoneally with 0.3 ml of 5 mg/ml of *Escherichia coli* LPS (Sigma-Aldrich, Saint Louis, USA) by dilution with phosphate-buffered saline (PBS) at room temperature. In tank 2, Int2 (*Vibrio*), a total of three fishes were injected intra-peritoneally with 0.3 ml of PBS dissolved culture pellet of *Vibrio harveyi* (∼e^8^ cell/ml) at room temperature. In tank 3, Int3 (PBS), used as control 1, three fishes received an intra-peritoneal injection of 0.3 ml of PBS for each fish. These fishes were not given access to feeds before sampling. Three fishes taken from the original freshwater tank were moved to tank 4, Int4 (FW;Feed), as control 2, containing 1000 l of freshwater. These fishes were fed twice daily with pelleted feed (Biomar, Nersac, France). Another three fishes from the original freshwater tank were moved to the freshwater tank 5, Int5 (FW;Fasting), and were not given access to feed before sampling. Three fishes in the seawater tank 6, Int6 (SW;Feed), were fed twice daily with pelleted feed before sampling. Three fishes from each of the tanks 1, 2 and 3 were sacrificed at ∼40 h post-challenges. Three fishes from each of the tanks 4, 5 and 6 were sacrificed at 8 days post-treatments. Intestine samples were taken from each fish of each tank and kept in Trizol reagent (Invitrogen, Carlsbad, USA) for RNA isolation.

### 2.2. Challenges and sampling for quantitative RT-PCR analysis

Eighteen seabass at the age of 11 months (body weight ∼330 g) originally maintained in a large tank containing 2000 l of freshwater were evenly divided into two tanks containing 1000 l of freshwater (Groups 1 and 2). Nine of the fishes in Group 1 were not given access to feed before sampling, and nine of the remaining fishes in Group 2 were fed twice daily with pelleted feed (Biomar, Nersac, France). Three fishes from each group were sacrificed at 8 days post-fasting. Intestine samples were taken for each fish and kept in Trizol reagent (Invitrogen, Carlsbad, USA) for RNA isolation.

For analysis of the functions of the splice variants of IFABP-a and -b genes, two extreme groups (i.e. smallest and biggest; *n* = 6/group) for body weight were selected from a population of ∼300 seabass at the age of 2 months. These fishes were originally maintained in a tank containing 2000 l of freshwater and were fed twice daily with pelleted feed (Biomar, Nersac, France). Intestine samples were taken for each fish and kept in Trizol reagent (Invitrogen, Carlsbad, USA) for RNA isolation.

### 2.3. RNA-seq sequencing

Total RNA from the intestine was isolated using the Trizol kit (Invitrogen, Carlsbad, USA). Total RNA from three fishes at each time point was equally mixed and submitted to Macrogen (Seoul, Korea) for RNA sequencing by using Roche/454 GS FLX Titanium platform. The total RNA quality was assessed using the Agilent 2100 Bioanalyzer. Ribosomal RNA was then removed prior to proceeding. cDNA rapid libraries were prepared according to the manufacturer's protocol (Roche, Central plaza, Singapore).

### 2.4. *De novo* assembly of the intestine transcriptome for the Asian seabass

GS FLX data were processed using the Roche GS FLX software (v 2.6). *De novo* assembly of transcriptome was carried out using the GS *De Novo* Assembler (v 2.6) with default assembly parameters. Singleton cleaning was performed with software SeqClean (http://sourceforge.net/projects/seqclean/) and Lucy (http://lucy.sourceforge.net/) with a minimum length of 100 bp.

### 2.5. Bioinformatics analysis

#### 2.5.1. Annotation and classification of transcripts

To assess the quality of the transcriptome assembly, 20 EST data sets were downloaded from NCBI databases and reciprocally compared with intestine transcriptome of Asian seabass using Blastn algorithm with an *E*-value threshold of *E*^−5^. These data sets included the EST data set (22 335 ESTs) of Asian seabass from NCBI, 15 intestine EST data (62 848 ESTs) and 4 unigene databases for the model fishes, *Gasterosteus aculeatus*, *Takifugu rubripes*, *Danio rerio* and *Oryzias latipes* (95 846 ESTs) (Supplementary Table S1). Batch blast of the unique sequences was carried out by using local Blast software (ver.2.2.25+) available from ftp://ftp.ncbi.nlm.nih.gov/blast/executables/blast+/LATEST/.

Singletons and isotigs from the *de novo* assembly were referred to as unique sequences to annotate them. They were blasted against GO database (http://www.geneontology.org/), Swissprot, Ref_protein and Refseq_RNA databases that retrieved from NCBI database using Blastn or Blastx algorithm with an *E*-value threshold of *E*^−5^.

#### 2.5.2. Annotation of pathways

Ortholog assignment and mapping of the unisequences to biological pathways were performed using the KEGG automatic annotation server (KAAS; http://www.genome.jp/tools/kaas/)^18^ with a threshold bit-score value of 40.

#### 2.5.3. Prediction of protein-coding regions and sequence alignment

OrfPredictor^19^ was used to predict protein-coding regions in EST-derived unisequences. The predicted protein sequences were then input into SignalP 4.0 server^20^ to predict potential signal peptide cleavage sites. Alignment of the predicted protein sequence for each splice variant was conducted using the online tool ClustalW2 (http://www.ebi.ac.uk/Tools/msa/clustalw2/).

#### 2.5.4. Identification of DEGs and significantly regulated pathways

Identification of DEGs from the count data was performed using the NOISeq-sim program^21^ with parameters setting as *v* = 0.1, *k* = 1.5, and other as defaults. Normalization was conducted using the RPKM method.^22^ The identified DEGs were then blasted against the KAAS database to identify the regulated pathways involved. Comparison of the DEGs and pathways among the four challenges was carried out with Venn diagrams.^23^ The RPKM-normalized transcript counts for the top 60 most DEGs in each pairwise comparison were then converted to natural logs and analysed by Cluster 3.0 with parameters as hierarchical clustering, uncentered correlation, and complete linkage (http://bonsai.ims.u-tokyo.ac.jp/~mdehoon/software/cluster/software.htm#ctv) to construct a heat map. Scatter plots between pairwise data sets were drawn with geWorkbench platform (https://cabig.nci.nih.gov/community/tools/geWorkbench).

#### 2.5.5. qPCR analysis of gene expressions and functions of splice variants of IFABP genes

For quantitative RT-PCR (q-PCR) analysis to confirm gene expression revealed by RNA-seq, total RNA from the same group (experimental group: three fishes sampled at 8 days post-fasting challenge and the control group) were equally mixed. Around 1 µg of DNase I-treated total RNA was reverse transcribed to cDNA by M-MLV reverse transcriptase (Promega, Madison, USA) with random hexamer primer as RT primer following the manufacturer's protocol. The resulting single-strand cDNA was assayed as DNA template by qPCR using primers for 56 genes from the RNA-seq data and the EF1A gene as the control (Supplementary Table S2). PCR in triplicates was performed with the KAPA™ SYBR® FAST qPCR Kits (Kapa Biosystems, Boston, USA) as described by the manufacturer, and an iQ5 PCR-machine (Bio-Rad, CA, USA). The ΔΔCT method was used for the analysis of the gene expression, and the values of triplicate RT-PCR reactions were normalized to EF1A gene expression.

The qRT-PCR analysis of expressions of the splice variants of two IFABP genes in the intestines of the Asian seabass were performed as described above with slight modifications. Briefly, to design primers for PCR, the exon/intron boundaries and divergence regions from the splice variants of IFABP genes were identified by using Sequencher 4.9 (GeneCodes, CA, USA). One primer pair that spans exon/intron boundary on the mRNA for each splice variant was designed using PrimerSelect (DNASTAR, DE, USA). This primer design allows differentiating the splice variants and does not amplify their genomic DNA. In addition, around 1 µg of DNase I-treated total RNA from each fish was reverse transcribed to cDNA. The resulting single-strand cDNA was assayed as DNA template by qPCR using primers for the six splice variants. The EF1A gene was used as a control (Supplementary Table S2).

### 2.6. Statistical analysis

The correlation coefficients between pairwise data sets were calculated by using the SAS (SAS Institute, Cary, USA). A *P*-value for significant difference was calculated using the *t*-test module installed in the Microsoft Office Excel 2008 program with parameters setting as two-tailed distribution and two-sample equal variance.

## 3. Results and discussion

### 3.1. *De novo* assembly and annotation of an intestine transcriptome for the Asian seabass

#### 3.1.1. Transcriptome sequencing by using 454 technology and *de novo* assembly

A total of 1 004 081 raw sequencing reads with an average length of 516 bp were generated by sequencing 6 cDNA libraries. The high-quality reads generated are available at the NCBI SRA browser (Run accession number: DRR002185-DRR002190). The *de novo* assembly produced a total of 83 911 unique sequences (referred to as unisequences) including 33 191 isotigs and 50 720 singletons (Table 1). The average size of isotigs was 992 bp and the N50 isotig size was 1191 bp, while the average length of the unisequences was 747 bp (Supplementary Fig. S1a and b). Compared with other *de novo* transcriptomes in fish, such as spleen transcriptome of the orange-spotted grouper with an average length of 504–547 bp,^24^ our study produced a much better transcriptome assembly. Contigs and singletons are also available upon request.

**Table 1.** Summary of *de novo* assembly of the intestine transcriptome of Asian seabass based on RNA-seq data

Library name	Int1 (LPS)	Int2 (Vibrio)	Int3 (PBS)	Int4 (FW;Feed)	Int5 (FW;Fasting)	Int6 (SW;Feed)	Total
Total read count	256 448	118 560	128 005	238 098	130 721	132 249	1 004 081
Total bases	130 465 540	62 854 483	64 921 797	122 920 944	69 894 007	65 705 252	516 762 023
Average read length (bp)	508.66	530.15	507.18	516.39	534.68	496.83	515.65
Assembled reads	196 136	100 981	108 743	188 846	108 392	104 819	831 771
Singleton number	26 696	7576	9204	19 682	9954	10 196	62 892
Isogroup number	12 001	6950	6230	9430	6442	6113	29 850
Isotig number	12 794	7278	6555	9951	6839	6471	33 191
Number of bases in isotigs	11 040 446	5 568 944	5 174 010	8 494 883	5 452 152	5 317 174	32 924 919
Average isotig size (bp)	862.94	765.18	789.32	853.67	797.22	821.69	991.98
N50 isotig size (bp)	964	781	831	953	834	902	1191
Largest isotig size (bp)	10 249	10 221	10 243	10 241	10 266	10 247	10 251
Number of singletons	22 091	7141	8522	16 480	9468	9402	50 720

LPS, lipopolysaccharide; PBS, phosphate-buffered saline; SW, seawater; FW, freshwater.

By using Blastn search reciprocally, only 46.46% (38 992) of the unisequences in the new built had significant matches to the Asian seabass NCBI EST data set. The remaining 44 919 (53.54%) unisequences without significant similarity to the previous EST data set were considered to represent new sequences in Asian seabass. We also compared the new assembly with the unigene sequences of the model fish species to evaluate the depth and breadth of our unisequence data set. 40.82 and 67.41% of transcriptome data sets of *Danio rerio* and *Gasterosteus aculeatus* had significant hits in the seabass intestine transcriptome, respectively. However, only 6.63–17.00% unisequences of the intestine transcriptome could be mapped onto the unigenes sequences of *Takifugu rubripes* and *Gasterosteus aculeatus*, respectively. These data suggest that a large number of new gene sequences are discovered in the Asian seabass intestine transcriptome. This intestine transcriptome can serve as a reference sequence database for intestine comparative transcriptome analysis in fish. The summary of reciprocal comparison between public EST data sets and the intestine transcriptome data set of the Asian seabass by Blastn program is presented in Supplementary Table S1.

#### 3.1.2. Gene annotation and functional classification

We found that 16 512 (49.75%; Refseq_RNA) to 18 036 (54.34%; Refseq_protein) isotig-originated unisequences, and 9915 (19.55%; Swissprot) to 28 861 (56.90%; refseq_RNA) singletons had significant matches to the different databases (Supplementary Table S3). A total number of 10 790 isotigs and 5501 singletons showed significant matches in 303 pathways. Metabolic pathways (735 unigenes), pathways in cancer (186 unigenes) and biosynthesis of secondary metabolites (155 unigenes) were the three pathways containing most unigenes. Supplementary Table S4 presents the KEGG automatic annotation of the Asian seabass intestine transcriptome. Combining of all these Blast results revealed that 20 318 isotigs (61.22%) and 32 037 singletons (63.16%) were annotated. The new intestine transcriptome supplies substantial new gene resources for studying interesting biological questions besides stress responses in fish.

#### 3.1.3. Prediction of ORFs

ORFs for 32 920 isotigs (99.18%) and 50 336 singletons (99.24%) were successfully predicted, suggesting that most unisequences without Blastx hits are derived from protein-coding genes. Based on the predicted protein sequences, 2140 signal peptides for isotigs and 1622 signal peptides for singletons were predicted using the SignalP 4.0 server (Supplementary Table S5). Because a signal peptide directs the transport of a protein, therefore, ∼4.48% of the transcriptome proteins were likely destined to the secretory pathway in the fish intestine. Our predictions are similar to the published data of other species, such as parasites (3305–4246 sequences; 6.5–6.9%)^25^ and trematode (1534 sequences; 5.03%).^26^

#### 3.1.4 Verification of differential expressions of genes by qPCR

To validate the digital gene expression analysis from the intestine RNA-seq data, 56 genes were randomly selected for qPCR analysis of samples at 8 days post-fasting challenge and the control. In the two data sets, most genes showed similar responses to the fasting challenge. The RNA-seq data had a positive linear relationship with qPCR data (Pearson correlation coefficients (*r*) = 0.58; Supplementary Fig. S2). There was no statistically significant difference (*t*-test: *P* = 0.21) between two data sets, which was in concordance with the report in channel catfish RNA-seq study.^13^ These results indicate that the RNA-seq technique could provide an excellent tool for expression profiling studies. For non-model organisms without reference genome and transcriptome sequences, although RNA-seq with the Illumina sequencing platform could generate a large number of short sequence reads, it is very difficult to assemble and analyse the reads. Therefore, 454 sequencing generating much longer reads is the suitable choice in Asian seabass. It is to note that due to a lower number of reads in 454 sequencing in comparison with the Illumina sequencing, some extremely low expression genes might not have been detected in this study. In the future, RNA-seq with the Illumina sequencing technology could be used for more detailed analysis of profiles of gene expression.

### 3.2. Stress responses in the Asian seabass intestine

#### 3.2.1. Identification of DEGs and regulated pathways in response to challenges

The interactions between stressors and stress responses are highly complex, and some stress responses may themselves act as stressors and vice versa.^2^ To obtain a general view of DEG expression patterns, pairwise comparison of expression abundance in the RNA-seq data sets was first conducted. The scatter plots showed a linear relationship between pairwise data sets with positive Pearson correlation coefficients (*r* = 0.82 to 0.99, *P* < 0.01) (Supplementary Fig. S3), suggesting that expression patterns of most genes are similar, and only a small portion of genes were expressed differentially among different stresses.

**Figure 1.**
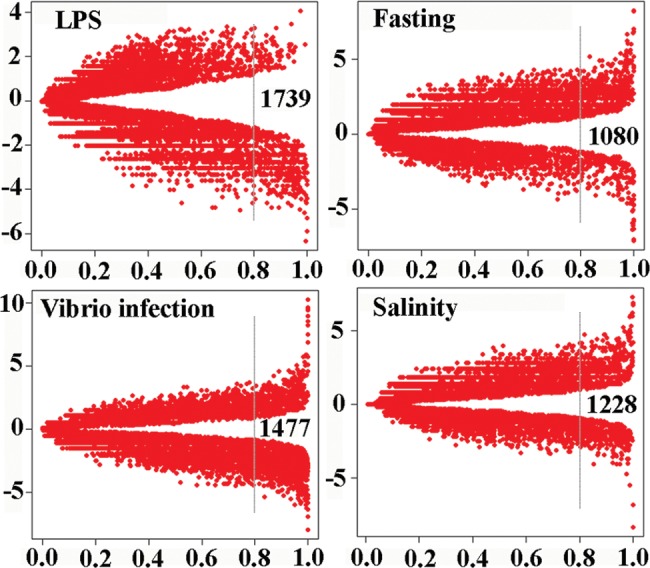
Volcano plots showing the gene expression differences among challenges and controls. The RPKM-normalized transcript count data sets were analysed by using the NOIseq program: the *x*-axis shows the probability for each gene of being differentially expressed and the *y*-axis shows the log-ratio (gene expression fold change after challenge). For each treatment, the total DEG number that identified by the program (probability 0.8 as a threshold) was shown.

To explore DEGs in response to different challenges, we performed statistical analysis of gene expression between pairwise samples using the NOISeq procedure. Because the fishes used in this analysis are heterogeneous populations, more restrictive parameters than the defaults in the software were applied during data analysis. Volcano plots in Fig. 1 show gene expression differences between challenges and controls. The numbers of DEGs in response to LPS and *Vibrio* infection were higher than those induced by fasting and high-salinity stressors. These results suggest that some molecular response mechanisms are dependent on the nature of the stress signals, and LPS and *Vibrio harveyi* infection are the stronger stressors among the four different challenges (i.e. LPS, *Vibrio harveyi*, high salinity and fasting).

When using probability 0.8 (means that the feature is four times more likely to be differentially expressed than non-differentially expressed) as the threshold for each gene of being differentially expressed, we found 1080 (Fasting) to 1739 (LPS) genes were differentially expressed between treatments and the respective controls (Table 2). Of which, 601 (high salinity) to 1139 (LPS) DEGs were down-regulated, and 410 (Fasting) to 627 (high salinity) DEGs were up-regulated. The observed values for DEGs are similar to those in the previous study based on RNA-seq in channel catfish, in which, 1633 DEGs at 3, 24 h, and 3 days between challenged and control intestine samples were revealed following *Edwardsiella ictaluri* infection.^13^ The DEGs in this study are similar to those in catfish.^13^ For example, around half of the DEGs in the category Cytoskeletal/Muscle Fiber Dynamics that were detected in channel catfish can be found in our DEG category following *Vibrio* infection, such as *Actin (cytoplasmic 1)*, *AHNAK nucleoprotein* and *Annexin A2*. These observations suggest that some physiological responses to stressors in fish are well conserved evolutionarily. Around 31% (*Vibrio* infection) to 50% (LPS injection) of the DEG sequences in this study had no hit in the current protein or RNA databases, suggesting that these genes are novel. These newly identified DEGs are of interest for further study. The list of DEGs that were identified using NOISeq (probability 0.8 as a threshold) is presented in Supplementary Table S6.

**Table 2.** Summary of the DEGs and regulated pathways that identified in response to four treatments in Asian seabass

Treatment	LPS injection	Vibrio infection	Fasting	High salinity
Total DEGs	1739	1477	1080	1228
Up-regulated	600	456	410	627
Down-regulated	1139	1021	670	601
Total regulated pathways	238	233	220	216
Up-regulated	160	178	158	168
Down-regulated	209	210	178	180

To illustrate the differential expression of genes detected in the intestine among different challenges, a heat map of RPKM-normalized transcript counts for the top 60 DEGs in each pairwise comparison was generated through hierarchical clustering analysis (Supplementary Fig. S4). Interestingly, these genes were generally down-regulated after challenges. For example, after LPS injection, we found that 57 of 60 genes were down-regulated. The down-regulated DEG category includes some important elements in immunity and defence response, such as, *Complement C1q-like* (≥4-fold), *Cathepsin L* (−32.7-fold), and *Heat shock 70 kDa protein 12A* (−12-fold). Similar results were also observed for *Vibrio* infection and during fasting (see details in Supplementary Fig. S4). The down-regulation of genes related to immunity and defence response may suggest that stressors depress the functions of some immune-related genes in the intestine. The significantly down-regulated genes after all four challenges may be used as biomarkers for detecting stresses in fish.

The DEGs were mapped to different KEGG pathways. The results of mapping to the DEGs are presented in Supplementary Fig. S5 and Supplementary Table S7a. Ortholog assignment and mapping of the DEGs to the biological pathways revealed that 216 (high salinity) to 238 (LPS) pathways were in response to the 4 treatments, respectively (Table 2 and Supplementary Table S7a). Many pathways previously found acting in neurodegenerative disorder (e.g. Parkinson's disease, Alzheimer's disease, Huntington's disease), regulation of cellular energy metabolism and biosynthesis (e.g. oxidative phosphorylation, cardiac muscle contraction, protein processing in the endoplasmic reticulum, fatty acid metabolism), and signal transduction (Peroxisome proliferator-activated receptor [PPAR] signalling pathway, chemokine signalling pathway, MAPK signalling pathway) responded to the four stressors, suggesting their important functions in stress response. Interestingly, metabolic pathways contain the most abundant DEGs whereby most of the genes were down-regulated after the four challenges. For instance, *glyoxylate/hydroxypyruvate reductase* and *arylamine N-acetyltransferase* were down-regulated under LPS and *Vibrio* injection; *3-hydroxyacyl-CoA dehydrogenase* and *ubiquinol-cytochrome c reductase cytochrome b subunit* were down-regulated after fasting and high-salinity treatments, respectively. The down-regulation of genes in metabolic pathways suggests that the four stressors inhibit metabolic functions of fishes. Ribosome biogenesis is among the most energy-consuming cellular processes,^27^ and it is therefore not surprising that the ribosome pathway is tightly controlled upon salinity change (significantly down-regulated by 7-fold), due to the disrupted homeostasis. Mature ribosomes are rapidly degraded by autophagy upon nutrient starvation in *Saccharomyces cerevisiae*.^27^ To our surprise, the number of up-regulated DEGs in the ribosome pathway, e.g. *large subunit ribosomal proteins L14e*, *L18Ae*, *L19e* and *L22e*, significantly increased by 23-fold, when compared with the number of down-regulated DEGs after fasting, implying that selective biosynthesis of ribosomes is functionally important in response to fasting in fish.

#### 3.2.2. Interaction among DEGs and regulated pathways in response to different stressors

**Figure 2.**
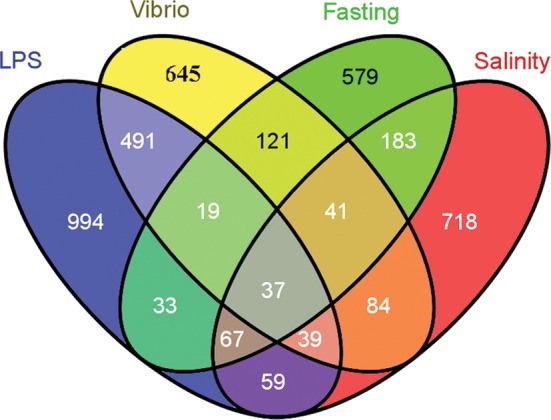
A Venn diagram describing overlaps among genes differentially expressed after treatments. All the DEGs (probability 0.8 as a threshold) under four treatments were compared with each other. The unique genes in each library and crosstalk genes among different libraries were illustrated.

Crosstalks between biotic and abiotic stress signalling have been extensively reported in plants,^28,29^ yeast and animals.^30^ We observed that a lot of DEGs responded to two or more treatments, which may indicate that gene interactions or shared pathways are involved in these stress responses. A Venn diagram in Fig. 2 describes the overlap among genes differentially expressed after different stress challenges. One hundred and twenty-one genes reacted to both *Vibrio* infection and fasting, and 37 genes were found differentially expressed in all 4 treatments. For example, aquaporins that was involved in osmoregulation^31^ and pathogen infection^32^ showed responses to all four challenges. The overlapping of a set of DEGs in response to all the four different stressors suggests that there is a global coordination for stress responses in the fish intestine.

**Figure 3.**
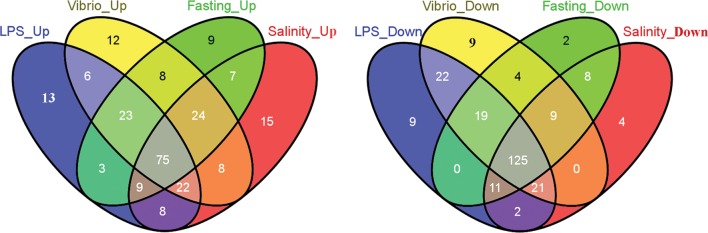
Venn diagrams describing overlaps among differentially regulated pathways after four treatments. The regulated pathways in response to four treatments were divided into two groups (up and down), and within each group, the regulated pathways were compared with each other. The overlapping pathway numbers among different libraries are illustrated.

To analyse the overlaps among differentially regulated pathways involved in the responses, we further compared the differences of regulated DEGs in each pathway under different conditions (Fig. 3 and Supplementary Table S7b). We found that the DEGs in 75 pathways showed significant up-regulation, and the DEGs in 125 pathways displayed significant down-regulation in response to all challenges. Sixty-two pathways were classified into the down-regulated pathway category after all treatments. These pathways were mainly involved in metabolism, digestion and absorption processes, e.g. vitamin digestion and absorption, mineral, fatty acid metabolism, nitrogen, beta-alanine, propanoate, pyruvate, fat, glycerolipid, and signalling pathways, e.g. GnRH, neurotrophin, Fc epsilon RI, TGF-beta, phosphatidylinositol and PPAR. Our data suggest that stresses not only inhibit the functions of metabolic pathways, but also repress signalling pathways responsible for reproductive hormone production and growth. Twelve unique pathways were specifically enriched in the up-regulated category after all treatments. These pathways are mainly associated with disorders, signalling transduction involved in innate defences and related processes encoding antimicrobial peptides and proteins, e.g. measles, pertussis, hepatitis C, lysosome, Toll-like receptor (TLR) signalling pathway, notch signalling pathway, NOD-like receptor signalling pathway and cytokine–cytokine receptor interaction. These results suggest that the four stressors induced the expression of genes that related to innate immune defences. Recent studies on mouse revealed that acute and short-term hormonal stress induced an early increase of immune cells in rats^33^ and other stresses significantly enhanced the fish immune response.^34^ These results supply evidence supporting that stresses enhanced some immune functions for a short time.^35^ However, it is not known why the functions of some immune-related genes were enhanced, while other immune-related genes were repressed, which is worthy to further study. PPAR signalling is known to increase in the liver after fasting^36^ and is down-regulated in the rat gastrocnemius muscle^37^ and in the human skeletal muscle^38^ after a 48-h fasting. In our study, down-regulation of gene expression in the PPAR signalling pathway upon LPS, *Vibrio* infection, conditions of fasting and high-salinity treatments suggests crosstalks for the PPAR signalling pathway among different conditions.

Challenges of fish with stressors can cause specific changes in gene regulation. About 43.67% (645 of 1477; *Vibrio* infection) to 58.47% (718 of 1228; high salinity) of the DEGs were differentially expressed in a treatment-specific manner, respectively (Fig. 2). These data imply the importance of their roles in response to specific stressors. For example, we observed *Annexin A2* (isotig00837), *Monoamine oxidase gene* (contig00009), *Butyrophilin subfamily 1 member A1* (isotig00448) and *Alkaline phosphatase* (isotig00361) are unique in response to LPS, infection, fasting and high salinity, respectively. Similar functions have been shown for these genes in previous studies. *Annexin* participates in the establishment of inflammation and the immune response.^13,39^ *Monoamine oxidase* expression in rats reduced after infection with *Pneumococcus*.^40^ *Butyrophilin subfamily 1 member A1* is assumed to contribute to milk secretion and lipid droplets,^41^ and *Alkaline phosphatase* is involved in osmotic stress.^42^ In addition, 9 (fasting) to 15 (high salinity) regulated pathways were treatment-specifically up-regulated, and 2 (Fasting) to 9 (*Vibrio*) regulated pathways were treatment-specifically down-regulated (Fig. 3). These unique pathways in response to different stressors generally contained few DEGs (less than four) (Supplementary Table S7b). Little is known about the expression response for most of these DEGs and pathways in fish during stress. The unique kinetic expression of specific genes and unique pathways implies their importance in host stress responses to specific stressors. It would be interesting to characterize their functions in future, such as how stressors are discriminated by host and the mechanisms involved in the stress response.

#### 3.2.3. The effects of stresses on the immune system of the intestine in fish

Although the intestine is not a major immune organ of teleost fish, it plays an importance role in defence against pathogen invasion.^43^ It is generally believed that stresses depress the functions of the immune system.^35^ Different stresses may have different impacts on immunity.^44^ Our intestine transcriptome data set allowed exploring effects of stress on the immune system of the intestines in fish. We found that many DEGs (i.e. 177, 244, 108, and 123 DEGs in response to LPS, *Vibrio* infection, fasting, and high-salinity challenges, respectively) were classified into the immune system process. These DEGs including interleukin-1, interleukin-8, transforming growth factor, antimicrobial enzymes (e.g. lysozyme, lectin), MHC-related proteins, immunoglobulin superfamily members and pathways such as lysosome and the TLR signalling pathway responding to infection have been discovered (Supplementary Tables S6 and S7a). These data suggest a dramatic stress-related immune response to stressors in fish intestines. Interestingly, most immune-related DEGs were down-regulated after challenges (see Supplementary Table S6), suggesting that these stresses partially repress the immune function of the intestine.

Fine-tuning of many immune-related DEGs were observed in response to different stressors. Stress induces an increase of a few inflammatory cytokines, such as C–C motif chemokine 25-like significantly increased after *Vibrio* infection, fasting, and salinity challenges. However, some other cytokines such as tumor necrosis factor ligand superfamily member 10-like, granulins-like, tumor necrosis factor receptor superfamily member 14-like were significantly down-regulated after the *Vibrio* infection. The members in the complement system, helping destroy the pathogens and eliminate the infection, also showed differential regulations. For example, complement C3-like is significantly increased after LPS challenge, which is an expected response to acute bacterial infection. However, complement C1q-like protein 2-like showed significant down-regulation after LPS and *Vibrio* challenges, but substantial up-regulation after fasting and salinity challenges. Our data suggest complicated regulation of cytokines and complement factors in the fish intestines during different stresses. Meprins generate biologically active IL-1β from its precursor pro-IL-1β and play a critical role in the inflammatory response.^45^ We found that meprin A subunit beta-like was significantly down-regulated (∼ 6–9-fold) after the *Vibrio* infection, but increased by 2–3-fold after fasting and salinity challenges, suggesting its important role in different stress responses. Immunoglobulins play critical role in mucosal immunity, acting as the first line of adaptive humoral immune defence at mucosal surfaces in mammals. We found that immunoglobulin lambda-like polypeptide 1-like was down-regulated (2–3-fold) after LPS injection and *Vibrio* infection, but significantly increased by 2-fold after fasting, suggesting differential expression level of the adaptive immune system in fish in response to different stressors. Serum amyloid helps the immune system to recognize invasion by bacteria.^46^ In the liver of Atlantic salmon, serum amyloid A, complement factor B, and serotransferrin were decreased in expression following starvation, but serum amyloid A and complement factor increased after infection.^47^ In our study, serum amyloid P-component-like was highly induced after LPS injection (∼2.6-fold), fasting (∼23-fold), and salinity (∼156-fold) challenges, but down-regulated after *Vibrio* infection (>100-fold). These results suggest that although there is a global coordination of gene expressions under different stresses, there is a fine-tuning of many immune-related DEGs in response to different stressors. Future studies on DEGs related to immune-responses would help to elucidate the complex alterations of the immunological network under stress.

### 3.3. Structure and functional implication of splice variants of IFABP genes

**Figure 4.**
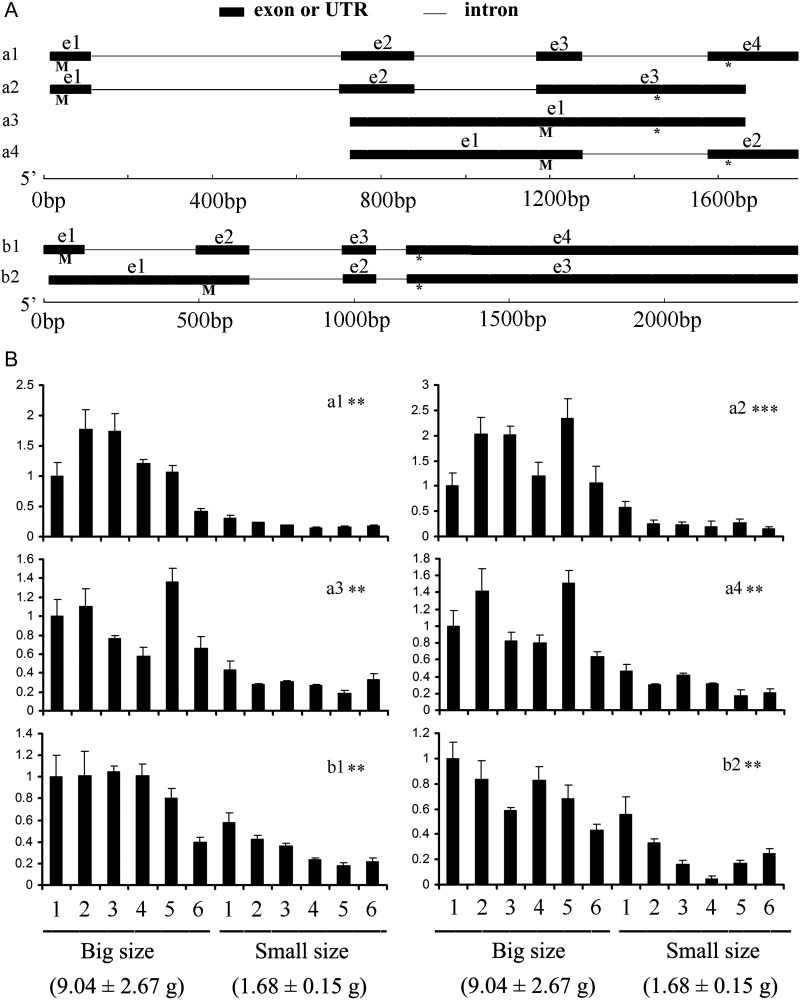
Alternative splicing variants of IFABP-a and -b genes and their expression in the intestines of the Asian seabass individuals with extreme growth traits. (A) The transcript sequences and their corresponding genomic sequences of the splice variants of IFABP-a and IFABP-b genes are presented. ‘e1’–‘e4’ show the exons and UTRs in variants, ‘M’ and ‘*’ denote the approximate location of the predicted translation start site and *stop codon for each variant*, respectively; (B) Gene expression of the splice variants in two groups of the Asian seabass with extreme body weight sizes. Significant level: ‘**’, *t*-test *P* value < 0.01 and ‘***’, *t*-test *P* value < 0.001.

Splice variants can increase the proteome diversity and cellular function.^48^ In the RNA-seq data, ∼7% genes contain splice variants (data not presented). Of which, IFABP (fatty acid-binding protein, intestinal) genes showed significant responses to different challenges. Since these challenges, such as fasting, seriously affect the nutritional and health status of fish, we speculated that IFABP genes are important to the growth and development of fish. To explore the functions of the IFABP genes, we cloned the genomic DNA regions of the IFABP genes. In comparison of the genomic DNA regions with the transcript sequences and the prediction of ORF for these genes, we identified four splice variants (a1-a4) for IFABP-a and two splice variants (b1-b2) for the IFABP-b gene (Fig. 4A and Supplementary Fig. S6). The length for the possible ORF for each variant ranged from 43 amino acids (a4) to 177 amino acids (a2). All of the predicted protein sequences showed sequence conservation with the reported IFABP genes. To find the association of the genes to growth traits, we developed SNP markers from their genomic sequences and conducted QTL mapping by using an Asian seabass F_2_ family with 359 individuals and their growth trait data.^49^ IFABP-a and IFABP-b were mapped onto the linkage group LG5 and LG14 of the Asian seabass genetic map, respectively. Interestingly, we found that an IFABP-a-SNP1245 marker was located near or on a QTL with a proportion of explained phenotypic variance of 10.2% for body weight that were measured at the age of 9 months.^49^ IFABP-a-SNP1245 showed high heterozygosity (CG genotype; 77–90%) in the large body weight group (*n* = 30) and showed low heterozygosity (20–30%) in the small body weight group (*n* = 30) (Fisher's exact test: *P* < 0.001).^49^ Both analyses suggested that the SNP mutation IFABP-a-SNP1245 was significantly associated with growth. To further characterize the expression pattern of IFABP splice variants, we performed the qPCR gene expression of the splice variants in the intestines of the Asian seabass samples with extreme growth traits (Fig. 4B). We found that all of the four splice variants of the IFABP-a gene and the two splice variants of the IFABP-b gene were significantly highly expressed in the big size group of fishes (*P* < 0.01), although there were small expression differences among individuals within same groups. The IFABP genes were abundantly expressed in the fish intestines^50,51^ and involved in the uptake of dietary fatty acids and their intracellular transport.^50^ In mammals, significant associations were found among fatness, the abundance of IFABP and growth.^52,53^ The present findings that growth was related to the IFABP expression in fish were consistent with previous researches.^52,53^ The higher ability to harvest dietary fatty acids from food as reflected by higher expression of IFABP genes in the intestines of big size fishes may partially explain their fast growth. Our study underlines the importance of splicing variants of IFABP genes in the growth and stress response of the Asian seabass.

### 3.4. Conclusions

This study represents the first RNA-seq-based gene expression analysis in response to several stressors in cultured food fish species, and supplies large transcriptome data for the addressing of biological questions in fish. We detected a broad representation of stress-related genes in many pathways in response to different challenges. The overlap among DEGs and regulated pathways after different challenges suggest a global coordination in response to different stressors. The differences in DEGs and regulated pathways in the response to distinct types of stress suggest the fine-tuning of gene regulation during different stresses. In addition, we detected dramatic immune responses of the intestine under different challenges. This study is the first step towards achieving a comprehensive view of the molecular mechanisms underlying stress responses. Further detailed analysis of the functions of DEGs and pathways, as well as their interactions will enhance our understanding of the molecular mechanisms involved in the response of fish to stressors.

## Supplementary data

Supplementary Data are available at www.dnaresearch.oxfordjournals.org.

## Funding

This research is supported by the National Research Foundation Singapore under its Competitive Research Program (CRP Award No. NRF-CRP002-001).

## Supplementary Material

Supplementary Data

## References

[1] Roelofs D., Aarts M.G.M., Schat H., van Straalen N.M. (2008). Functional ecological genomics to demonstrate general and specific responses to abiotic stress. Funct. Ecol..

[2] Harper C., Wolf J.C. (2009). Morphologic effects of the stress response in fish. ILAR J..

[3] Barton B.A. (2002). Stress in fishes: a diversity of responses with particular reference to changes in circulating corticosteroids. Integ. Comp. Biol..

[4] von Borell H. (2001). The biology of stress and its application to livestock housing and transportation assessment. J. Anim. Sci..

[5] Marioni J.C., Mason C.E., Mane S.M., Stephens M., Gilad Y. (2008). RNA-seq: an assessment of technical reproducibility and comparison with gene expression arrays. Genome Res..

[6] Esteve-Codina A., Kofler R., Palmieri N., Bussotti G., Notredame C., Pérez-Enciso M. (2011). Exploring the gonad transcriptome of two extreme male pigs with RNA-seq. BMC Genomics.

[7] Ordas A., Hegedus Z., Henkel C.V. (2011). Deep sequencing of the innate immune transcriptomic response of zebrafish embryos to *Salmonella* infection. Fish Shellfish Immunol..

[8] Utsumi Y., Tanaka M., Morosawa T. (2012). Transcriptome analysis using a high-density oligomicroarray under drought stress in various genotypes of cassava: an important tropical crop. DNA Res..

[9] Huang J., Lu X., Yan H. (2012). Transcriptome characterization and sequencing-based identification of salt-responsive genes in *Millettia pinnata*, a semi-mangrove plant. DNA Res..

[10] Torres T.T., Metta M., Ottenwälder B., Schlötterer C. (2008). Gene expression profiling by massively parallel sequencing. Genome Res..

[11] Edwards C.E., Parchman T.L., Weekley C.W. (2012). Assembly, gene annotation and marker development using 454 floral transcriptome sequences in *Ziziphus celata* (Rhamnaceae), a highly endangered, Florida endemic plant. DNA Res..

[12] Woo S., Yum S., Kim D.W., Park H.S. (2009). Transcripts level responses in a marine medaka (*Oryzias javanicus*) exposed to organophosphorus pesticide. Comp. Biochem. Physiol. C. Toxicol. Pharmacol..

[13] Li C., Zhang Y., Wang R. (2012). RNA-seq analysis of mucosal immune responses reveals signatures of intestinal barrier disruption and pathogen entry following *Edwardsiella ictaluri* infection in channel catfish, *Ictalurus punctatus*. Fish Shellfish Immunol..

[14] Yue G.H., Li Y., Chao T.M., Chou R., Orban L. (2002). Novel microsatellites from Asian sea bass (*Lates calcarifer*) and their application to broodstock analysis. Mar. Biotechnol..

[15] Tan S.L., Mohd-Adnan A., Mohd-Yusof N.Y., Forstner M.R., Wan K.L. (2008). Identification and analysis of a prepro-chicken gonadotropin releasing hormone II (preprocGnRH-II) precursor in the Asian seabass, *Lates calcarifer*, based on an EST-based assessment of its brain transcriptome. Gene.

[16] Xia J.H., Yue G.H. (2010). Identification and analysis of immune-related transcriptome in Asian seabass *Lates calcarifer*. BMC Genomics.

[17] Xia J.H., He X.P., Bai Z.Y., Lin G., Yue G.H. (2011). Analysis of the Asian seabass transcriptome based on expressed sequence tags. DNA Res..

[18] Moriya Y., Itoh M., Okuda S., Yoshizawa A., Kanehisa M. (2007). KAAS: an automatic genome annotation and pathway reconstruction server. Nucl. Acids Res..

[19] Min X.J., Butler G., Storms R., Tsang A. (2005). OrfPredictor: predicting protein-coding regions in EST-derived sequences. Nucleic Acids Res..

[20] Bendtsen J.D., Nielsen H., von Heijne G., Brunak S. (2004). Improved prediction of signal peptides: signalP 3.0. J. Mol. Biol..

[21] Tarazona S., García-Alcalde F., Dopazo J., Ferrer A., Conesa A. (2011). Differential expression in RNA-seq: a matter of depth. Genome Res..

[22] Mortazavi A., Williams B.A., McCue K., Schaeffer L., Wold B. (2008). Mapping and quantifying mammalian transcriptomes by RNA-Seq. Nat. Methods.

[23] Oliveros J.C. (2007). http://bioinfogp.cnb.csic.es/tools/venny/index.html.

[24] Huang Y., Huang X., Yan Y. (2011). Transcriptome analysis of orange-spotted grouper (*Epinephelus coioides*) spleen in response to Singapore grouper iridovirus. BMC Genomics.

[25] Young N.D., Campbell B.E., Hall R.S. (2010). Unlocking the transcriptomes of two carcinogenic parasites, *Clonorchis sinensis* and *Opisthorchis viverrini*. PLoS Negl. Trop. Dis..

[26] Young N.D., Jex A.R., Cantacessi C. (2011). A portrait of the transcriptome of the neglected trematode, *Fasciola gigantica*-biological and biotechnological implications. PLoS Negl. Trop. Dis..

[27] Kraft C., Deplazes A., Sohrmann M., Peter M. (2008). Mature ribosomes are selectively degraded upon starvation by an autophagy pathway requiring the Ubp3p/Bre5p ubiquitin protease. Nat. Cell Biol..

[28] Fujita M., Fujita Y., Noutoshi Y. (2006). Crosstalk between abiotic and biotic stress responses: a current view from the points of convergence in the stresssignaling networks. Curr. Opin. Plant Biol..

[29] AbuQamar S., Luo H., Laluk K., Mickelbart M.V., Mengiste T. (2009). Crosstalk between biotic and abiotic stress responses in tomato is mediated by the AIM1 transcription factor. Plant J..

[30] Kultz D. (2005). Molecular and evolutionary basis of the cellular stress response. Annu. Rev. Physiol..

[31] Laforenza U., Cova E., Gastaldi G. (2005). Aquaporin-8 is involved in water transport in isolated superficial colonocytes from rat proximal colon. J. Nutr..

[32] Guttman J.A., Samji F.N., Li Y., Deng W., Lin A., Finlay B.B. (2007). Aquaporins contribute to diarrhoea caused by attaching and effacing bacterial pathogens. Cell. Microbiol..

[33] Dhabhar F.S., Malarkey W.B., Neri E., McEwen B.S. (2012). Stress-induced redistribution of immune cells—from barracks to boulevards to battlefields: a tale of three hormones—Curt Richter Award winner. Psychoneuroendocrinology.

[34] Tort L. (2011). Stress and immune modulation in fish. Dev. Comp. Immunol..

[35] Herbert T.B., Cohen S. (1993). Stress and immunity in humans: a meta-analytic review. Psychol. Med..

[36] Yoon J.C., Puigserver P., Chen G. (2001). Control of hepatic gluconeogenesis through the transcriptional coaotivator PGC-1. Nat. Biotechnol..

[37] de Lange P., Farina P., Moreno M. (2006). Sequential changes in the signal transduction responses of skeletal muscle following food deprivation. FASEB J..

[38] Tsintzas K., Jewell K., Kamran M. (2006). Differential regulation of metabolic genes in skeletal muscle during starvation and refeeding in humans. J. Physiol..

[39] Culhane J.F., Rauh V.A., Goldenberg R.L. (2007). Stress, bacterial vaginosis, and the role of immune processes. Curr. Infect. Dis. Rep..

[40] Popenenkova Z.A., Andreeva D.A. (1966). Monoamine oxidase activity of rat organs during experimental pneumococcus infection. Bull. Exp. Biol. Med..

[41] Ogg S.L., Weldon A.K., Dobbie L., Smith A.J.H., Mather I.H. (2004). Expression of butyrophilin (Btn1a1) in lactating mammary gland is essential for the regulated secretion of milk-lipid droplets. PNAS.

[42] Lim C., Ozkanca R., Flint K.P. (1996). The effects of osmotic stress on survival and alkaline phosphatase activity of *Aeromonas hydrophila*. FEMS Microbiol. Letters.

[43] Klostermeier U.C., Barann M., Wittig M. (2011). A tissue-specific landscape of sense/antisense transcription in the mouse intestine. BMC Genomics.

[44] Dorin Dragoş D., Tănăsescu M.D. (2010). The effect of stress on the defense systems. J. Med. Life.

[45] Herzog C., Haun R.S., Kaushal V., Mayeux P.R., Shah S.V., Kaushal G.P. (2009). Meprin A and meprin alpha generate biologically functional IL-1beta from pro-IL-1beta. Biochem. Biophys. Res. Comm..

[46] Yuste J., Botto M., Bottoms S.E., Brown J.S. (2007). Serum amyloid P aids complement-mediated immunity to *Streptococcus pneumoniae*. PLoS Pathog..

[47] Martin S.A.M., Douglas A., Houlihan D.F., Secombes C.J. (2010). Starvation alters the liver transcriptome of the innate immune response in Atlantic salmon (*Salmo salar*). BMC Genomics.

[48] Furnham N., Ruffle S., Southan C. (2004). Splice variants: a homology modeling approach. Proteins.

[49] Xia J.H., Lin G., He X. (2013). Whole genome scanning and association mapping identified a significant association between growth and a SNP in the IFABP-a gene of the Asian seabass. BMC Genomics.

[50] Chen X., Shi Z. (2009). Sequence analysis of the full-length cDNA and protein structure homology modeling of FABP2 from *Paralichthys Olivaceus*. Bioinform. Biol. Insights.

[51] Sharma M.K., Denovan-Wright E.M., Degrave A. (2004). Sequence, linkage mapping and early developmental expression of the intestinal-type fatty acid-binding protein gene (fabp2) from zebrafish (*Danio rerio*). Comp. Biochem. Physiol., Part B: Biochem. Mol. Biol..

[52] Woudstra T.D., Drozdowski L.A., Wild G.E. (2004). The age-related decline in intestinal lipid uptake is associated with a reduced abundance of fatty acid-binding protein. Lipids.

[53] Fawcett G.L., Roseman C.C., Jarvis J.P. (2008). Genetic architecture of adiposity and organ weight using combined generation QTL analysis. Obesity.

