# Supplementary material for: Analysis of Stress-Responsive Transcriptome in the Intestine of Asian Seabass (Lates calcarifer) using RNA-Seq
Source: DNA Res. 2013 Jun 10;20(5):449–60. doi: 10.1093/dnares/dst022 (PMC3789556; doi:10.1093/dnares/dst022)
Supplement: Supplementary Data [file supp_20_5_449__index.html]

Analysis of Stress-Responsive Transcriptome in the Intestine of Asian Seabass (Lates calcarifer) using RNA-Seq — Supplementary Data 

# Analysis of Stress-Responsive Transcriptome in the Intestine of Asian Seabass (*Lates calcarifer*) using RNA-Seq

## 

Supplementary Data

**Files in this Data Supplement:**

- Supplementary Figure 1 - tif file
- Supplementary Figure 2 - tif file
- Supplementary Figure 3 - tif file
- Supplementary Figure 4 - xls file
- Supplementary Figure 5 - tif file
- Supplementary Figure 6 - tif file
- Supplementary Table 1 - xls file
- Supplementary Table 2 - xls file
- Supplementary Table 3 - xls file
- Supplementary Table 4 - xls file
- Supplementary Table 5 - xls file
- Supplementary Table 6 - xls file
- Supplementary Table 7 - xls file
