# Supplementary figures and images for: Analysis of Stress-Responsive Transcriptome in the Intestine of Asian Seabass (Lates calcarifer) using RNA-Seq
Source: DNA Res. 2013 Jun 10;20(5):449–60. doi: 10.1093/dnares/dst022 (PMC3789556; doi:10.1093/dnares/dst022)

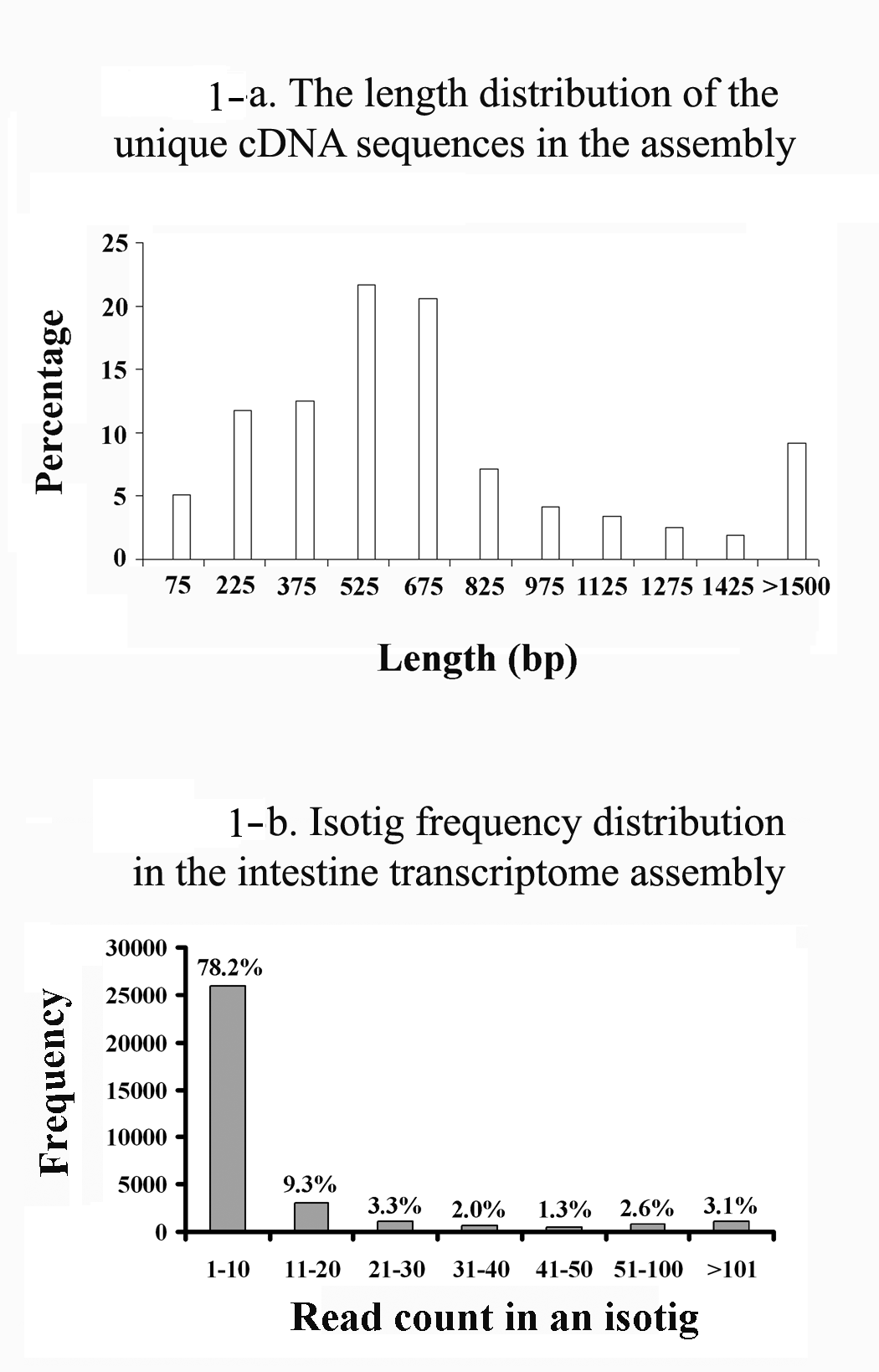

Supplement: Supplementary Data [file supp_dst022_dst022supp_fig1.tif]

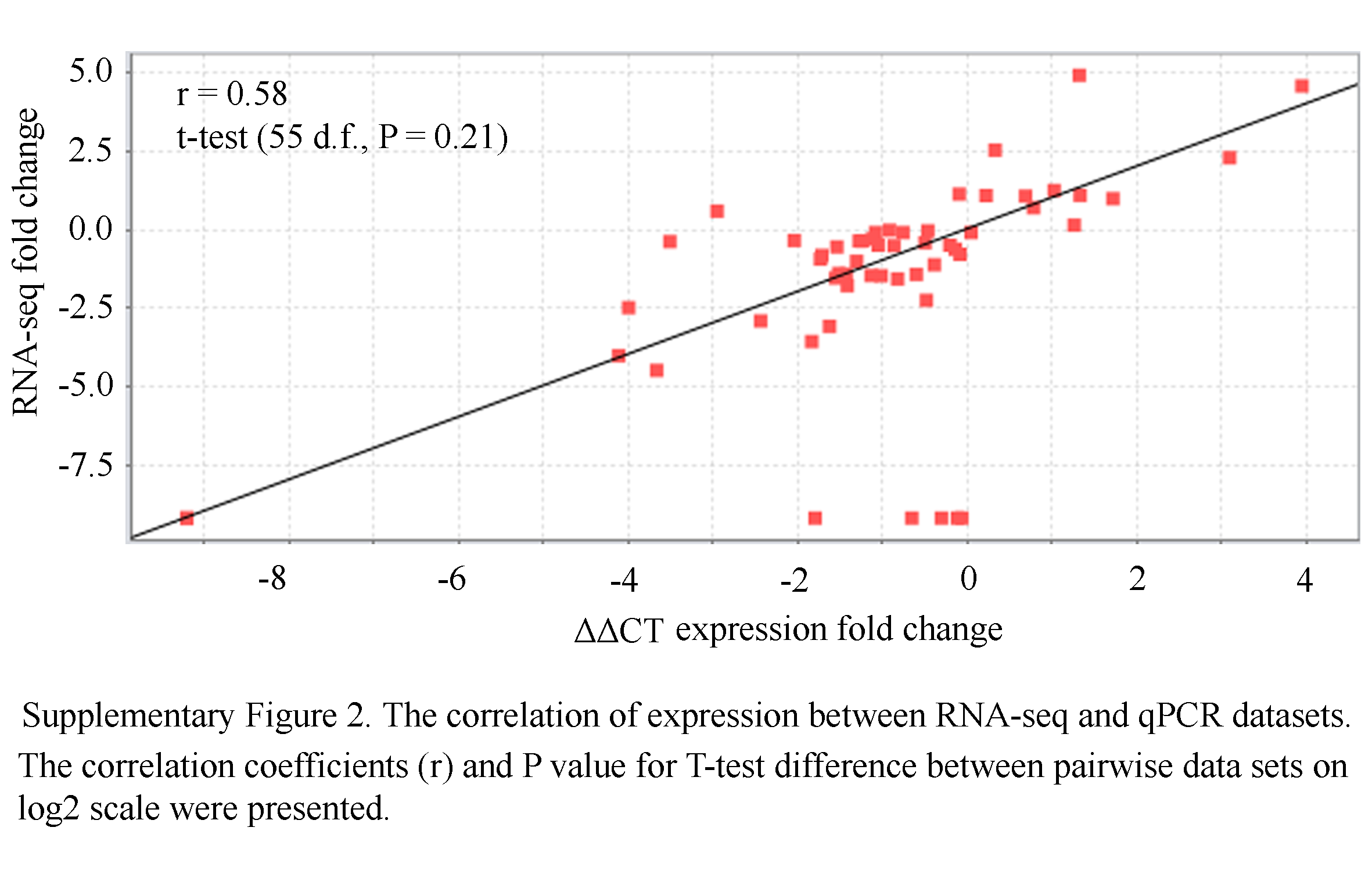

Supplement: Supplementary Data [file supp_dst022_dst022supp_fig2.tif]

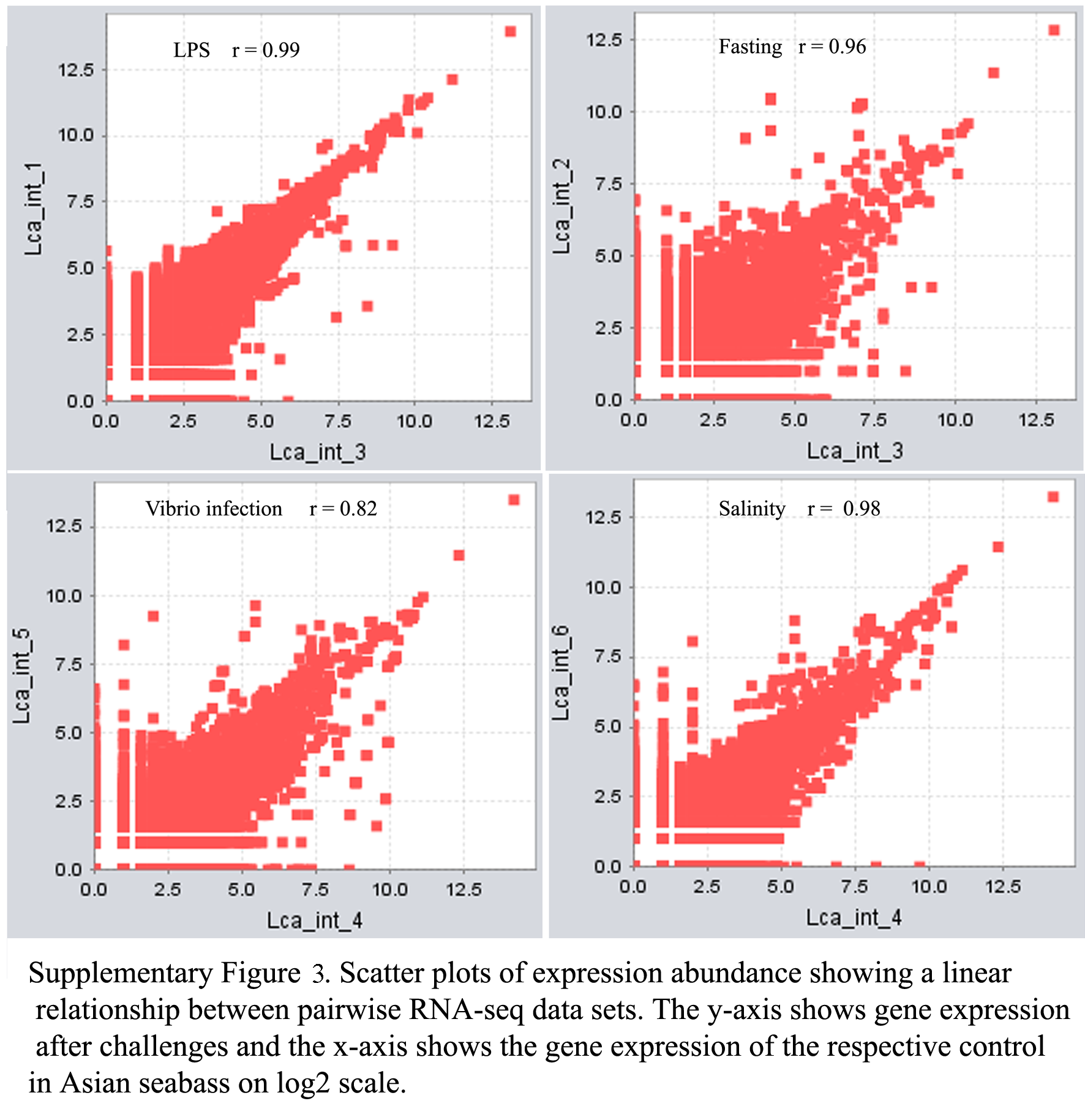

Supplement: Supplementary Data [file supp_dst022_dst022supp_fig3.tif]

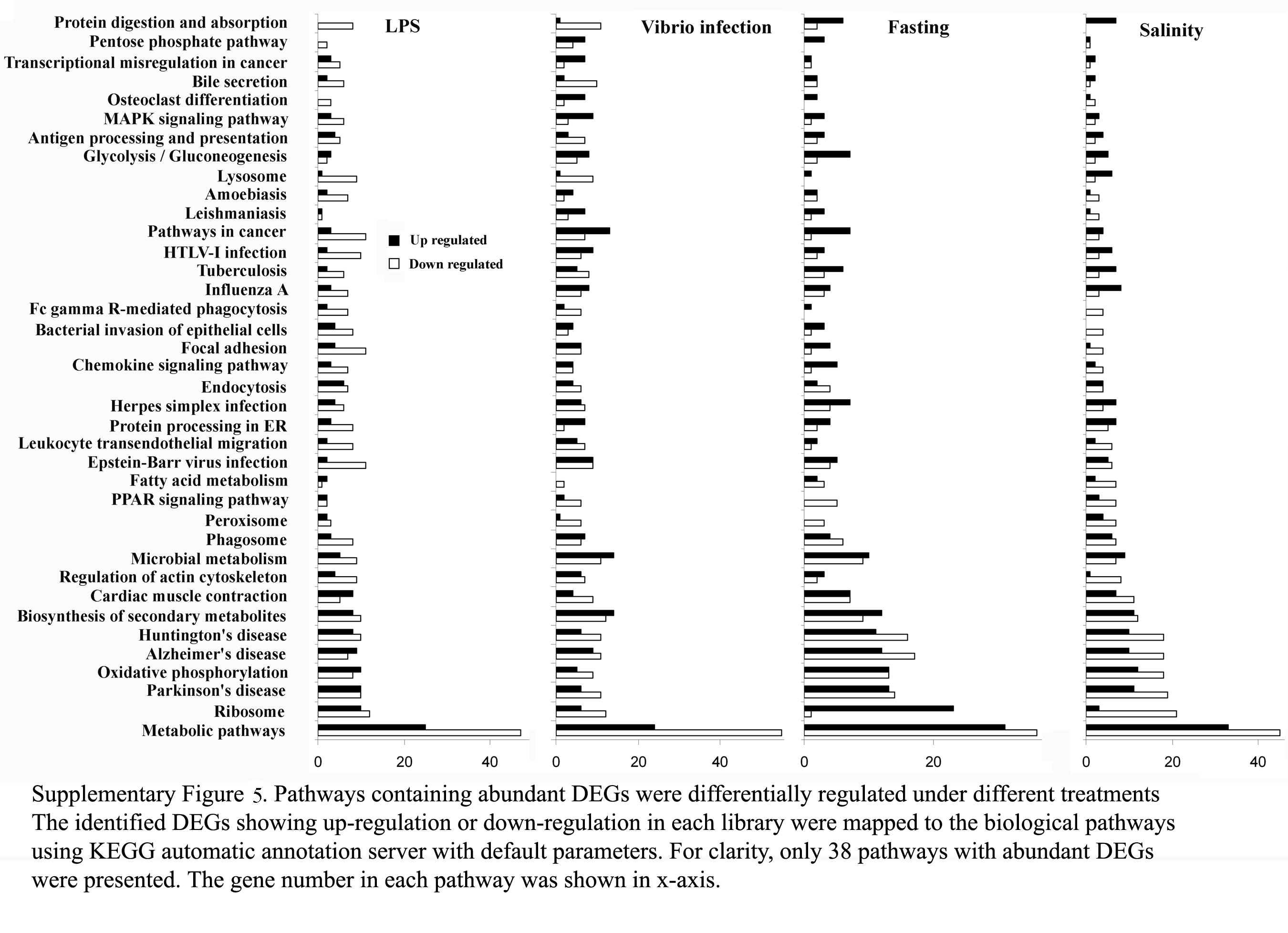

Supplement: Supplementary Data [file supp_dst022_dst022supp_fig5.tif]

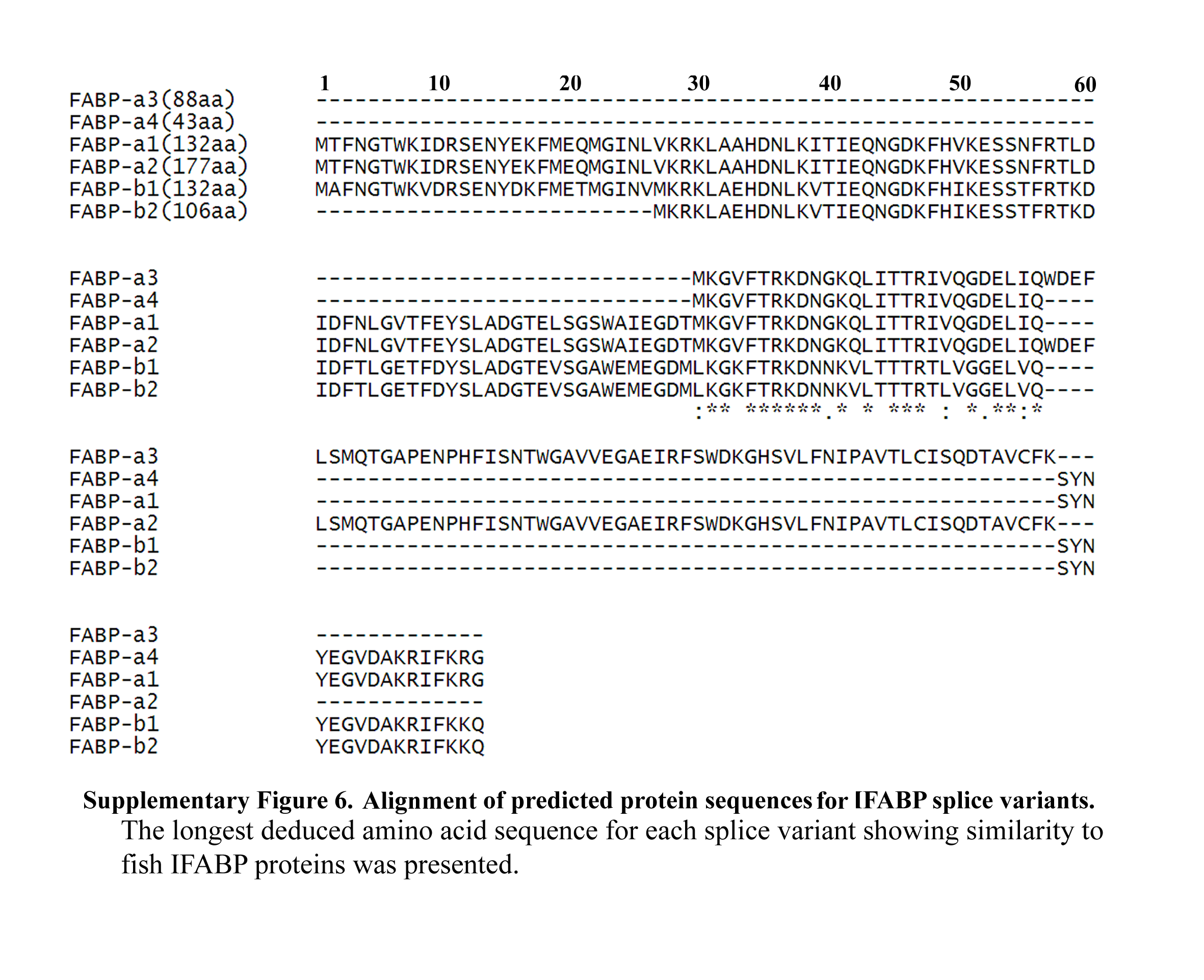

Supplement: Supplementary Data [file supp_dst022_dst022supp_fig6.tif]
